# The effect of miscellaneous oral dosage forms on the environmental pollution of sulfonamides in pig holdings

**DOI:** 10.1186/s12917-016-0688-6

**Published:** 2016-04-01

**Authors:** Jessica Stahl, Katrin Zessel, Jochen Schulz, Jan Henrik Finke, Christel Charlotte Müller-Goymann, Manfred Kietzmann

**Affiliations:** Department of Pharmacology, Toxicology and Pharmacy, University of Veterinary Medicine Hannover, Foundation, Buenteweg 17, 30559 Hannover, Germany; Institute for Animal Hygiene, Animal Welfare and Farm Animal Behaviour, Bischofsholer Damm 15, 30173 Hannover, Germany; Institut Pharmazeutische Technologie, Technische Universität Braunschweig, Mendelssohnstr. 1, 38106 Braunschweig, Germany

**Keywords:** Dust, Sulfonamides, Environmental pollution, Antibiotic residues, Pigs

## Abstract

**Background:**

Due to antibiotic treatment of humans and animals, the prevalence of bacterial resistances increases worldwide. Especially in livestock farming, large quantities of faeces contaminated with antibiotics pose a risk of the carryover of the active ingredient to the environment. Accordingly, the aim of the present study was the evaluation of the benefit of different oral dosage forms (powder, pellets, granula) in pigs concerning the environmental pollution of sulfadiazine. Two subtherapeutic dosages were evaluated in powder mixtures to gain information about their potential to pollute the pig barn. Furthermore, a new group of pigs was kept in the stable after powder feeding of another pig group to determine the possible absorption of environmentally distributed antibiotics.

Pigs were orally treated with three dosage forms. Simultaneously, sedimentation and airborne dust were collected and plasma and urine levels were determined.

**Results:**

All formulations result in comparable plasma and urine levels, but massive differences in environmental pollution (powder > pellets, granula). Pigs housing in a contaminated barn exhibit traces of sulfadiazine in plasma and urine.

**Conclusion:**

Using pharmaceutical formulations like pellets or granula, the environmental pollution of sulfonamides can significantly be diminished due to massive dust reduction during feeding.

## Background

To achieve an efficient treatment of animals and humans suffering from bacterial disease, an adequate antibiotic treatment is often required. A closer look into the antibacterial treatment of animals in Europe demonstrates that sulfonamides, tetracyclines and ß-lactam antibiotics are the most frequently used antibacterial agents [1–3]. Sulfonamides can be used in the treatment against gram-positive and gram-negative bacteria, as well as several rickettsia and protozoa (*Toxoplasma, Coccidia*) [4]. Active metabolites of sulfonamide excreted in urine or feces may pollute the environment [5–9]. Contamination from animal husbandry primarily affects soil and water, with pig, poultry and cattle manure, slurry and liquid dung, hospital waste water and sewage being the main sources [8, 10, 11]. Moreover, it has been demonstrated that sulfonamides contaminate ground water in particular by surface run off or sediment of shrimp ponds [12–15]. These antibiotic residues in the environment deriving from the antibiotic drug itself or its antibiotic metabolites pose the risk of the development of bacterial resistance due to subinhibitory concentrations (concentrations beyond the minimal inhibitory concentration) [16]. The antibiotic substance may be inhaled and swallowed. Furthermore, it can contaminate the skin of individuals and thus gets in contact with the normal skin flora, or it can orally be incorporated into the body, due to the contamination of food products. Tetracyclines and sulfonamides for example have been found in agricultural crops after distribution of antibiotic containing manure [17, 18]. Consequently, intestinal bacteria can be affected, as well. Another risk is, that antibiotics in stable dust can be distributed into the environment of the stable via emission [19–21].

Concerning the current problems of antibacterial resistances in both human and veterinary medicine and the associated problems of the inability of healing seriously ill individuals, it is of high importance to reduce the dissemination of antibacterial agents into the environment. Hamscher et al. (2003) demonstrated that antibiotic residues can be determined in stable dust for a long time [22]. Therefore, the present study was conducted to investigate the ability of different pharmaceutical formulations of antibiotics (sulfonamides) in the treatment of pigs to reduce environmental contaminations. Sulfonamide application was performed via powder, pellets and granula in order to test the absorption of the antibiotic agent in the oral dosage forms with various firmnesses (powder < pellets < granula). Environmental contamination was examined by collecting sedimentation dust in the stable and aerosol. Furthermore, untreated pigs (sentinels) were housed in a dry-cleaned stable after treatment of another pig group with sulfadiazine powder for several days to examine the effect of sulfonamide residues on plasma and urine samples of these untreated animals.

## Methods

### Sulfadiazine formulations

For powder manufacturing, usual pig feed (powder, Scharnebecker Muehle, Germany, comprising corn, wheat, barley, peas, oat and sorghum) was supplemented with sulfadiazine in the relevant concentrations (2.5 mg/kg body weight (BW), 5 mg/kg BW and 25 mg/kg BW) during a mixing process. Such sulfadiazine containing powder was also used for producing pellets in a pelletising machine under high pressure.

A granular formulation of sulfadiazine was produced via fluidized-bed spray-granulation. Potato starch was used as a binder in the granulation liquid consisting of 6 %(w/w) potato starch, 0.1 %(w/w) sulfadiazine and 93.9 %(w/w) deionized water. The granulation liquid was boiled for starch gelation. 4000 g of pig feed were placed in a fluidized-bed spray-granulator (WSG U5, Glatt GmbH) and sprayed at a spray rate of 40 rpm. After the spraying process, the granules were recovered and dried to a humidity of 7 % before filling and further application.

### Animals

24 female piglets of a hybrid breed (db. Viktoria × db.77), all of which weighing 10–15 kg at the beginning of the experiment, were obtained from the Lehr- und Forschungsgut Ruthe, Germany. The pigs were kept under the same conditions in groups of 6 animals and had free access to tap water from drinking nipples. The pen measured 23.52^2^, with 6 animals in bays of 8.8^2^. A ventilation system maintained a constant temperature of 19-20 ° C in the stable, which was verified by random measurements.

All pigs were clinically healthy (determined via visual examination) during the study. The study was approved by the LAVES File number 33.12-42502-04-11/0338.

### Experimental procedure

Pharmacokinetic studies were carried out in 4 groups of 6 pigs to determine the plasma and urine concentrations of sulfadiazine as well as the environmental pollution. Therefore, sulfadiazine was supplemented to the feed (powder mixing, pelletisation, granulation), which was given twice a day for four days. Events like feeding and cleaning were standardized. Sulfadiazine contamination of the stable was excluded before starting the experiment.

The whole experiment was performed in three different parts:

The first group of 6 pigs was treated via powder feeding (recommended formulation type) as follows: After 1 week of acclimatisation, the pigs were weight and received sulfadiazine (Trimosulf®, WDT, Garbsen, Germany) in powder in three different concentrations (two subtherapeutic concentrations) on 4 consecutive days. Between all treatments a washout-phase of three days was inserted (determined by a separate experiment). The first treatment was 2.5 mg/kg (subtherapeutic dosage) BW (body weight) for 4 days, followed by a washout-phase of three days. Afterwards, each pig received 5.0 mg/kg BW (subtherapeutic dosage) sulfadiazine for 4 days, followed by three non-medicated days. Finally, 25 mg/kg BW sulfadiazine (recommended dosage according to the package leaflet) was orally administered to the pigs for four days. Afterwards, the pigs were removed from the stable, which underwent a dry cleaning process and 6 untreated piglets moved into the stable in order to simulate a carry-over of sulfadiazine of a polluted stable. These untreated pigs were kept in the stable for 6 days and were fed with powder feed without antibiotic supplementation.

The second and third group of pigs was treated with sulfadiazine incorporated into pellets or granula in order to determine formulations of sulfadiazine with a high biocompatibility and low environmental contamination. The animals were treated with sulfadiazine (25 mg/kg BW) via pellets or granula over 4 days each. The sulfadiazine concentration in the feed was as followed: 94 mg/kg (powder; 2.5 mg/kg BW), 151 mg/kg (powder; 5 mg/kg BW), 820 mg/kg (powder, pellets, granula; 25 mg/kg BW).

### Chemicals

Sulfadiazine and sulfamerazine were purchased from Sigma-Aldrich (Steinheim, Germany) and were of the highest purity available. Methanol (HPLC-grade) was obtained from Applichem, Darmstadt, Germany.

### Determination of plasma concentrations

Plasma samples were taken immediately before antibiotic medication and after 3 h. Therefore, blood was collected from the jugular vein of each pig and was collected in EDTA-containing plastic tubes. After centrifugation for 10 min (3000 × g) the plasma was collected and stored at −20 ° C until extraction procedure. Blood of untreated pigs (sentinel pigs) was collected once a day 30 min after feeding.

**Determination of urine concentrations** Urine samples were collected spontaneously once a day of each pig and were stored at −20 ° C until analysis.

### Collection of sedimentation dust

**Fig. 1:**
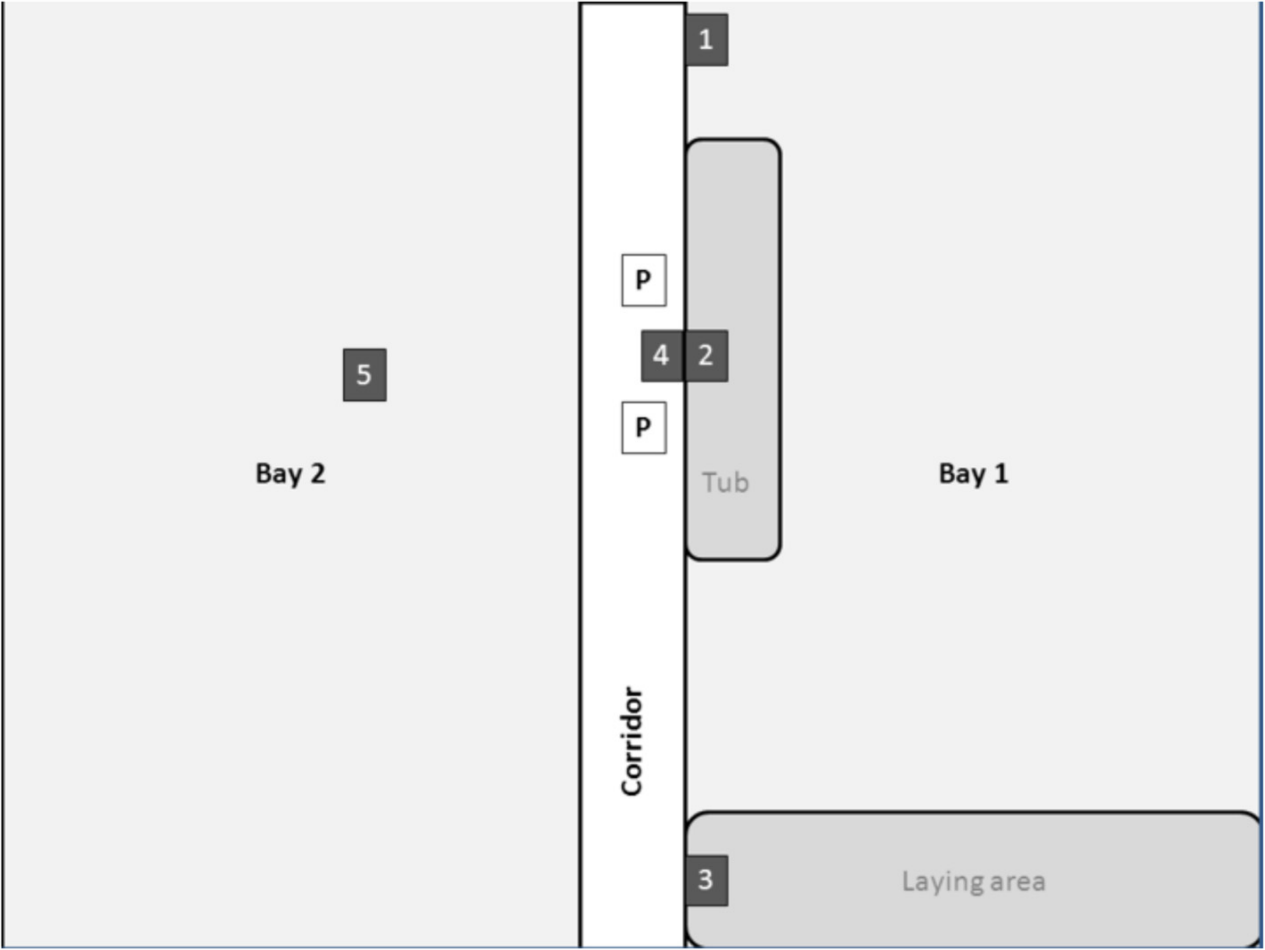
Scheme of the pig stable. The numbers indicate the 5 areas of sedimentation dust sample withdrawal (1. window sill, 2. grid above the tub, 3. grid above the lying area, 4. gutters in front of the tub and 5. bay barrier on the other side of the stable), and “P” shows the positions of air samplings. Bay 1 was used for all experimental setups

Sedimentation dust was collected at five different localisations in the stable and was stored at–20 ° C until extraction. Therefore, a card sheet was used to collect the dust from a predefined surface area and the dust was weight before extraction to determine the amount of sulfadiazine in μg/mg dust. The localisations are shown in Fig. 1.

The different areas are presented in Table 1, as well as height of the sampling area and the distance to the feeding area.

**Table 1:** Locations of the sedimentation dust samples withdrawal in the stable with distance to the tub and height of sampling area

No.	Location of the sampling areas	Vertical distance to the feeding tub (m)	Height of the sampling area (m)
1	window sill	2	2.1
2	grid above the tub	0.0	1.4
3	grid above the lying area	2	1.4
4	gutters in front of the tub	0.6	0.0
5	bay barrier in bay 2	2.5	1.1

### Aerosol collection

Air samples were taken simultaneously at two locations in the animal house (Fig. 1). Therefore SKC pumps with IOM samplers were used (PCWR (SKC INC., Eighty Four, P.A. USA). The air flow of the pumps was adjusted to 3 l/min. Dust was sampled on polycarbonate filters (0.8 μm diameter of the pores; Whatman, Dassel, Germany) over a time period of 9 h. The filters were weight before and after usage to determine the amount of collected dust.

### Extraction procedures

Sulfadiazine was extracted from plasma, urine, and stable dust according to van Duijkeren (1994) [23]. Sulfamerazine was used as internal standard for extraction procedure.

### Analytical methodology

High performance liquid chromatography (HPLC) was used to determine the amount of sulfadiazine and sulfamerazine consisting of the following parts (all Beckmann, Muenchen, Germany) according to Zessel et al. 2013 [24]. A 508-autosampler was used to inject 100 μl of the samples into the HPLC-system and a 126 Solvent System Pump maintained a constant flow of 1 ml/min. Separation was achieved on a Rp-18 LiChroCart 250-4-column (5 μm, 25 cm, Merck, Darmstadt, Germany) at 41 ° C. Detection was performed at 264 nm by a UV–VIS-166-Detector. The mobile phase consisted of 15 % methanol and 85 % McIlvaine-citrate buffer (pH 2.2). The limit of quantification (LOQ) was 0.1 μg/ml, the limit of detection was 0.09 μg/ml.

### Statistics

Statistical analyses were carried out with the GraphPad Prism program (version 6.05, GraphPad Software Inc., San Diego, CA, USA). A two way ANOVA was used to gain information about significance, which was defined as *p* < 0.05.

## Results

Sulfadiazine treatment via powder resulted in dust contaminations at all localisations in the stable with the highest amounts at the bay barrier in bay 2, followed by the tub (up to 1.7 μg/mg dust), the windowsill, the lying area, and the gutters in front of the tub (maximum 0.7 μg/mg dust, Table 2). Collected aerosol samples exhibited up to 8 μg sulfadiazine per m3 air during powder feeding of the recommended dosage (Table 3).

**Table 2:** Amount of sulfadiazine in μg/mg sedimentation dust at 5 localizations on 4 days

Localization	Treatment		μg sulfadiazine per mg sedimentation dust			
			Day 1	Day 2	Day 3	Day 4
1: Distance to the tub 2 m, height 2.10 m	Powder	2.5 mg/kg BW	< LOQ	< LOQ	< LOQ	< LOQ
		5.0 mg/kg BW	< LOQ	< LOQ	< LOQ	< LOQ
		25 mg/kg BW	0.8	1.2	1.1	1.3
	Pellets	25 mg/kg BW	< LOQ**	< LOQ**	< LOQ**	< LOQ**
	Granula	25 mg/kg BW	< LOQ**	< LOQ**	< LOQ**	< LOQ**
	Sentinels	untreated	1.3	1.9	1.0	1.1
2: Distance to the tub 0 m, height 1.40 m	Powder	2.5 mg/kg BW	< LOQ	< LOQ	< LOQ	< LOQ
		5.0 mg/kg BW	< LOQ	0.3	0.2	0.4
		25 mg/kg BW	0.6	1.3	1.8	1.7
	Pellets	25 mg/kg BW	< LOQ	< LOQ	< LOQ*	< LOQ*
	Granula	25 mg/kg BW	< LOQ	< LOQ	< LOQ*	< LOQ*
	Sentinels	untreated	1.3	1.3	1.3	1.4
3: Distance to the tub 2 m, height 1.40 m	Powder	2.5 mg/kg BW	< LOQ	< LOQ	< LOQ	< LOQ
		5.0 mg/kg BW	< LOQ	n.d.	0.1	0.1
		25 mg/kg BW	0.2	0.5	0.7	0.8
	Pellets	25 mg/kg BW	< LOQ	< LOQ	< LOQ*	< LOQ*
	Granula	25 mg/kg BW	< LOQ	< LOQ	< LOQ*	< LOQ*
	Sentinels	untreated	0.8	0.7	0.6	0.6
4: Distance to the tub 0.6 m, height 0 m	Powder	2.5 mg/kg BW	n.d.	< LOQ	< LOQ	n.d.
		5.0 mg/kg BW	0.1	0.2	0.1	0.1
		25 mg/kg BW	0.2	0.5	0.7	0.5
	Pellets	25 mg/kg BW	< LOQ	< LOQ	< LOQ*	< LOQ
	Granula	25 mg/kg BW	< LOQ	< LOQ	< LOQ*	< LOQ
	Sentinels	untreated	0.8	0.9	0.9	0.8
5: Distance to the tub 2.5 m, height 1.10 m	Powder	2.5 mg/kg BW	< LOQ	< LOQ	< LOQ	< LOQ
		5.0 mg/kg BW	< LOQ	< LOQ	< LOQ	0.1
		25 mg/kg BW	0.2	n.d.	0.9	2.8
	Pellets	25 mg/kg BW	< LOQ	< LOQ	< LOQ	< LOQ
	Granula	25 mg/kg BW	< LOQ	< LOQ	< LOQ	< LOQ
	Sentinels	untreated	0.9	1.1	1.0	1.0

*BW* body weight, *LOQ* limit of quantification; * = *p* < 0.05 (vs. pellets), ** = *p* < 0.01 (vs. pellets)

**Table 3:** Amount of sulfadiazine per m3 air (aerosol) measured on 4 days during the experiment

Aerosol		μg sulfadiazine per m3 air			
		Day 1	Day 2	Day 3	Day 4
Powder	2.5 mg/kg BW	< LOQ	< LOQ	< LOQ	< LOQ
	5.0 mg/kg BW	0.1	< LOQ	0.3	0.4
	25 mg/kg BW	2.7	8.0	5.7	3.2
Pellets	25 mg/kg BW	< LOQ	< LOQ*	< LOQ	0.2
Granula	25 mg/kg BW	< LOQ	0.2*	0.1	< LOQ
Sentinels	untreated	1.6	1.2	0.3	0.4

*BW* body weight, *LOQ* limit of quantification; * = *p* < 0.05 (vs. powder)

The ability of other oral dosage forms to diminish environmental contamination of sulfadiazine around treated pigs in comparison to powder feeding was examined in another experimental setup. Therefore, pellets and granula were manufactured. Sedimentation dust samples and aerosols (Tables 2 and 3) revealed amounts of sulfadiazine lower than the LOQ and maximum amounts of 0.1 to 0.2 μg/m3 air for both dosage forms. Even 10 % and 20 % of the recommended dosage given via powder feed, resulted in sulfadiazine contamination of the stable, that are sometimes even higher than dust samples collected during treatment with the recommended dosage given via pellets or granula.

**Fig. 2:**
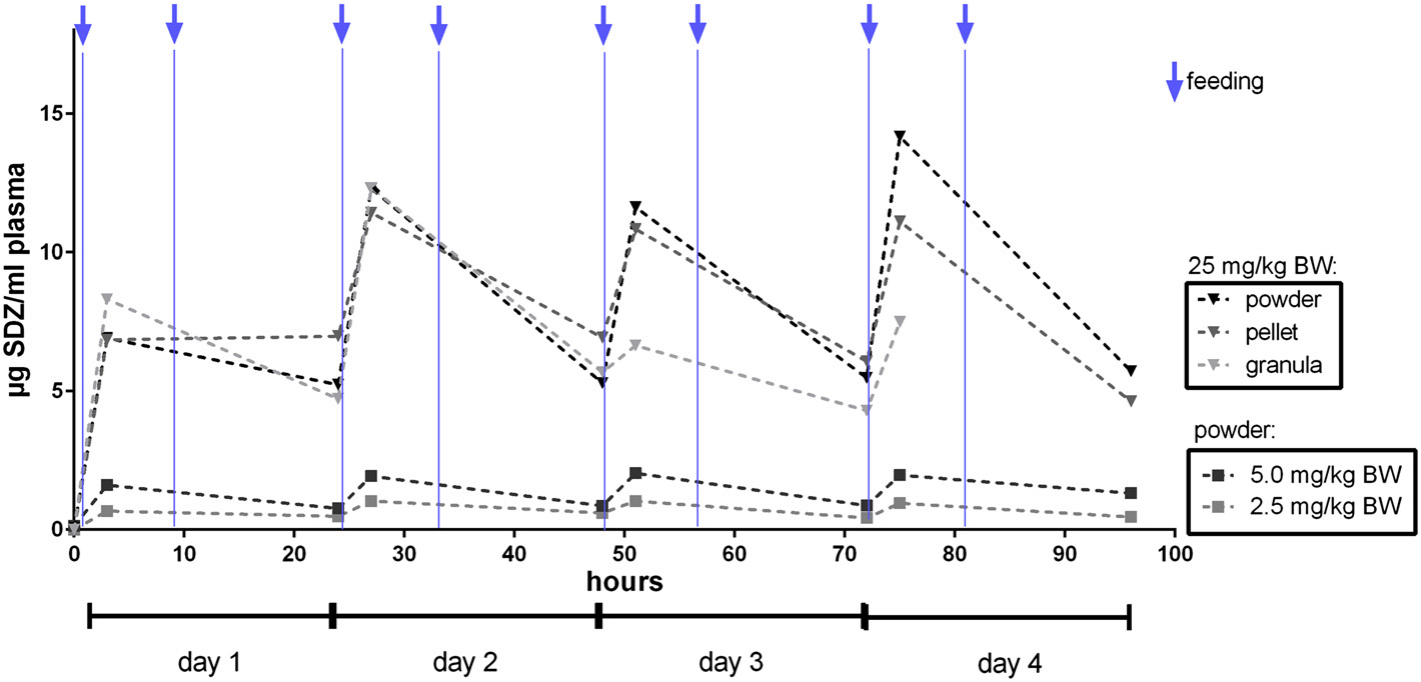
Plasma concentration of sulfadiazine (SDZ; mean) in six pigs after powder feeding of sulfadiazine in three dosages: 2.5 mg/kg BW (body weight; grey box), 5 mg/kg BW (black box) and 25 mg/kg BW (black triangle) and after pellet (dark grey triangle) and granula (bright grey triangle) feeding in a concentration of 25 mg/kg BW; feeding was performed twice daily (asterisks)

**Fig. 3:**
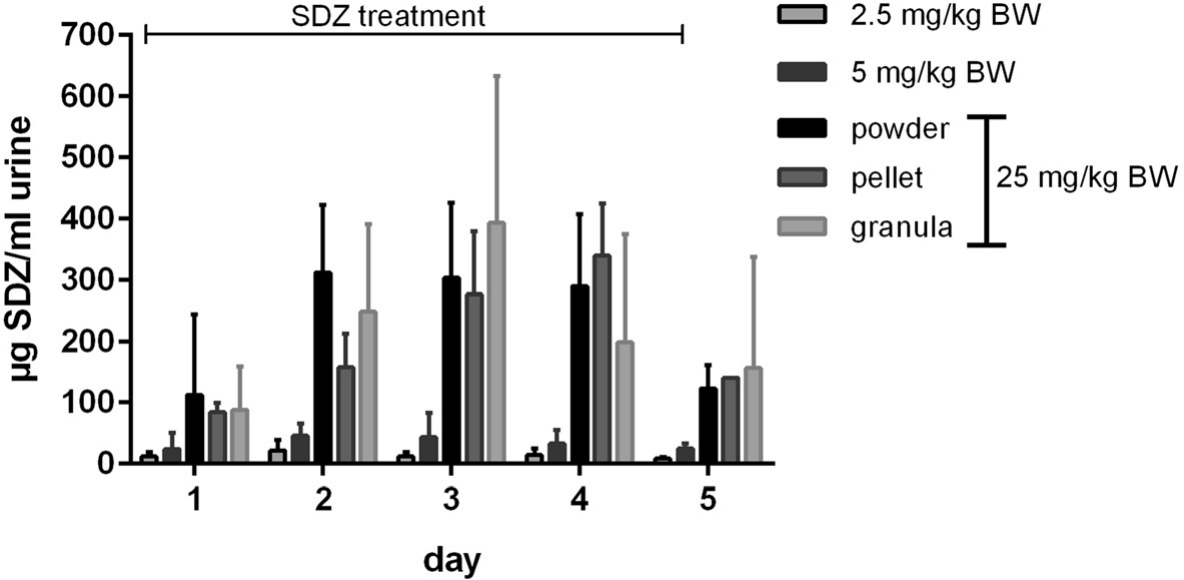
Urine concentration of sulfadiazine (SDZ; mean ± standard deviation) in six pigs after powder feeding of sulfadiazine in three dosages (2.5 mg/kg BW (body weight), 5 mg/kg BW and 25 mg/kg BW) and after pellet and granula feeding; feeding was performed twice daily on day 1–4

Plasma and urine samples of all treated animals revealed similar plasma and urine levels (maximum plasma levels up to 14.2 μg/ml and urine levels up to 393 mg/ml, Figs. 2 and 3) of all dosage forms.

**Fig. 4:**
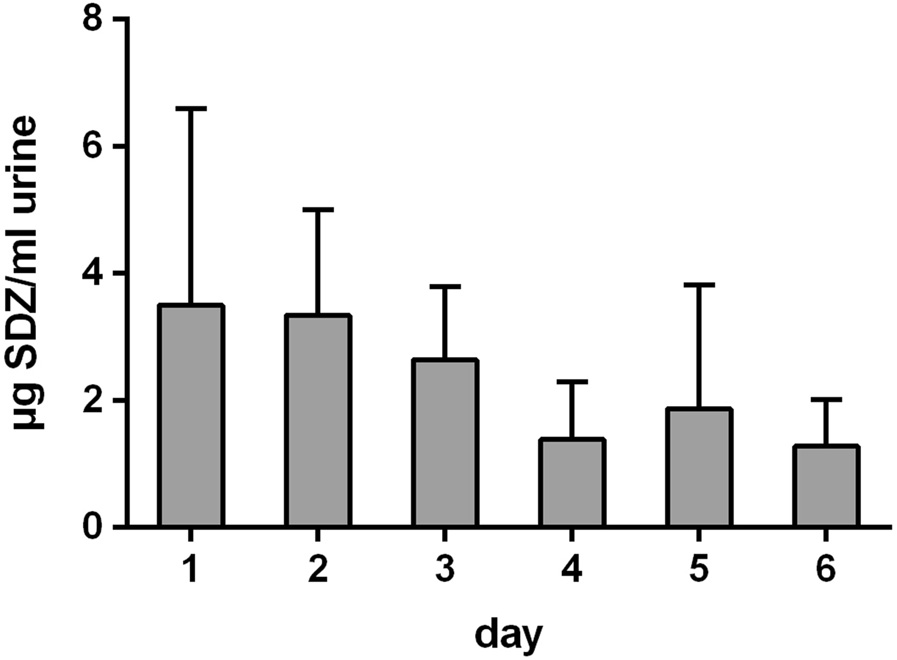
Urine concentration of sulfadiazine (SDZ) in six untreated pig, all of which were housed in the stable after removing orally treated pigs (25 mg/kg BW over four days) and after dry cleaning; data are shown as mean + standard deviation; LOQ = 0.1 μg/ml

Untreated pigs (sentinel animals) were kept in the stable after powder treatment of six other pigs for four days. Analysis of the plasma demonstrates that only small amounts of sulfadiazine are detectable in plasma (day 1 and 4 < LOQ) (Fig. 2), but with concentrations in the urine up to 3.5 μg/ml on the first day of housing in the stable (Fig. 4).

## Discussion

One aim of the present study was to examine the amount of environmental pollution of a commonly used oral dosage form (powder) of the test substance sulfadiazine in a pig stable during a four-day treatment in comparison to two other dosage forms. This general understanding is necessary since examinations of Hamscher et al. (2003) demonstrated that antibiotic residues (parent or metabolites) can be determined in stable dust during two decades [22]. Therefore, sulfadiazine powder was added to the powder feed, was mixed and was directly given to the pigs in a tub. The results of the dust samples demonstrate the importance of dust reduction in the stable of antibacterially treated animals, since sulfadiazine treatment via powder resulted in dust contaminations at all localisations in the stable. The difference in the degree of contamination in the different areas shows no satisfying correlation to the distance to the feeding area but may be explained by the air movement in the stable. Consequently, air movement resulting from the air conditioning has to be considered to reduce antibiotic contamination of untreated animals, but also the animals itself may be the reason for this differences, since trouble in the tub during feeding may lead to swirl up small dust particles containing sulfadiazine [21, 25].

The ability of other oral dosage forms to diminish environmental contamination of sulfadiazine around treated pigs in comparison to powder feeding was examined in a second experimental setup. Therefore, pellets and granula were manufactured. Sedimentation dust samples and aerosols revealed amounts of sulfadiazine lower than the LOQ (maximum amounts of 0.1 to 0.2 μg/m3 air) for both dosage forms. Even 10 % and 20 % of the recommended dosage given via powder feed, resulted in sulfadiazine contamination of the stable, that are sometimes even higher than dust samples collected during treatment with the recommended dosage given via pellets or granula.

Potential causes of this significantly lower sulfadiazine concentration in the environment of pellet and granula feeding in comparison to powder treatment have to be considered. First of all, the origin of dust has to be elucidated, since dust represents a mixture of particles deriving from the litter, the animals (skin, hair, faeces), and the food [21, 26]. The litter was the same in all experimental setups, so that this can be excluded as main reason for dust reduction. The animals had all the same background, age and weight and were housed under the same conditions, but since sulfadiazine is excreted mainly via the urine, the excretion rates caused by differences in the bioavailability of the different dosage forms had to be investigated. Therefore, plasma and urine samples of all treated animals were determined during the experiment and revealed similar plasma and urine levels for all dosage forms. Since all dosage forms exhibited nearly the same bioavailability, it can be concluded that the sulfadiazine distribution took place during the feeding process in dependence to the dosage form.

The possibility to absorb antibiotic residues from the environment was shown by Kietzmann et al. (1995) and raised the question if the detected sulfadiazine concentration in the environment during powder feeding is high enough to affect untreated animals in the dry cleaned stable. Therefore, untreated pigs (sentinel animals) were kept in the stable after powder treatment of six other pigs for four days. Analysis of the plasma demonstrates that only small amounts of sulfadiazine are detectable in plasma (day 1 and 4 < LOQ), but with concentrations in the urine up to 3.5 μg/ml on the first day of housing in the stable. This is not astonishing, since the stable was only dry cleaned and in sedimentation dust and aerosol samples exhibited sulfadiazine concentrations up to 1.9 μg/mg sedimentation dust and 1.6 μg/m3 air. To get knowledge about the magnitude of absorption, the comparison of the sentinel animals with the subtherapeutic dosages treated animals demonstrates that urine concentrations of the sentinel animals were 3–8 times lower than the sulfadiazine concentration in urine of the animals treated with 10 % of the recommended dosage.

The sentinel animals were exposed to a mean sedimentation dust concentration of 1.0 μg/mg (Table 2) and reached blood concentrations just below the limit of quantification (approximately 0.05-0.07 μg/ml, data not shown). To reach such a low plasma concentration the animals (body weight of 10–15 kg) had to orally adsorb 19–71 mg sedimentation dust (assuming a total blood volume of 4-7 % of the BW, [27] and 100 % oral bioavailability) from the environment. Since the aerosol exhibited mean sulfadiazine concentrations of 0.87 μg/m3 air (Table 3), a pig with a body weight of 10–15 kg and a blood concentration of 0.05-0.07 mg/ml had to absorb 20–73.5 μg sulfadiazine, which in turn is inhaled via 23–84 m3 air. Assuming a lung ventilation of 4–5.6 l/min. [28], with a bioavailability via the lungs of 100 %, 128–704 h would be necessary to absorb this amount of sulfadiazine via aerosol. Therefore, it can be concluded that the oral uptake of dust sedimentation from the stable represents the main source of absorption and may be slightly completed via the lungs.

## Conclusion

The present study demonstrates the importance of the pharmaceutical formulation on the entry of antibiotic residues into the environment of orally treated pigs. Pellets and granula produced lower concentrations of environmental contamination with sulfadiazine.

### Ethics approval

The study was approved by the LAVES File number 33.12-42502-04-11/0338.

### Availability of data and material

All datasets are available in the main manuscript.

## Abbreviations

EDTA: ethylenediaminetetraacetic acid
HPLC: high performance liquid chromatography
SDZ: sulfadiazine
SMR: sulfamerazine
